# ZnSe quantum dots modified with a Ni(cyclam) catalyst for efficient visible-light driven CO_2_ reduction in water†
†Electronic supplementary information (ESI) available: Experimental details, additional QD characterisation, control experiments, time-resolved spectroscopy. See DOI: 10.1039/c7sc04429a. Additional data related to this publication are available at the University of Cambridge data repository (https://doi.org/10.17863/CAM.17941).


**DOI:** 10.1039/c7sc04429a

**Published:** 2018-01-24

**Authors:** Moritz F. Kuehnel, Constantin D. Sahm, Gaia Neri, Jonathan R. Lee, Katherine L. Orchard, Alexander J. Cowan, Erwin Reisner

**Affiliations:** a Christian Doppler Laboratory for Sustainable SynGas Chemistry , Department of Chemistry , University of Cambridge , Lensfield Road , Cambridge CB2 1EW , UK . Email: reisner@ch.cam.ac.uk ; http://www-reisner.ch.cam.ac.uk; b Stephenson Institute for Renewable Energy , Department of Chemistry , The University of Liverpool , Crown Street , Liverpool L69 7ZD , UK . Email: A.J.Cowan@liverpool.ac.uk

## Abstract

A precious metal and Cd-free photocatalyst system for efficient CO_2_ reduction in water is reported. The hybrid assembly consists of ligand-free ZnSe quantum dots (QDs) as a visible-light photosensitiser combined with a phosphonic acid-functionalised Ni(cyclam) catalyst, NiCycP. This precious metal-free photocatalyst system shows a high activity for aqueous CO_2_ reduction to CO (Ni-based TON_CO_ > 120), whereas an anchor-free catalyst, Ni(cyclam)Cl_2_, produced three times less CO. Additional ZnSe surface modification with 2-(dimethylamino)ethanethiol (MEDA) partially suppresses H_2_ generation and enhances the CO production allowing for a Ni-based TON_CO_ of > 280 and more than 33% selectivity for CO_2_ reduction over H_2_ evolution, after 20 h visible light irradiation (*λ* > 400 nm, AM 1.5G, 1 sun). The external quantum efficiency of 3.4 ± 0.3% at 400 nm is comparable to state-of-the-art precious metal photocatalysts. Transient absorption spectroscopy showed that band-gap excitation of ZnSe QDs is followed by rapid hole scavenging and very fast electron trapping in ZnSe. The trapped electrons transfer to NiCycP on the ps timescale, explaining the high performance for photocatalytic CO_2_ reduction. With this work we introduce ZnSe QDs as an inexpensive and efficient visible light-absorber for solar fuel generation.

## Abstract 2

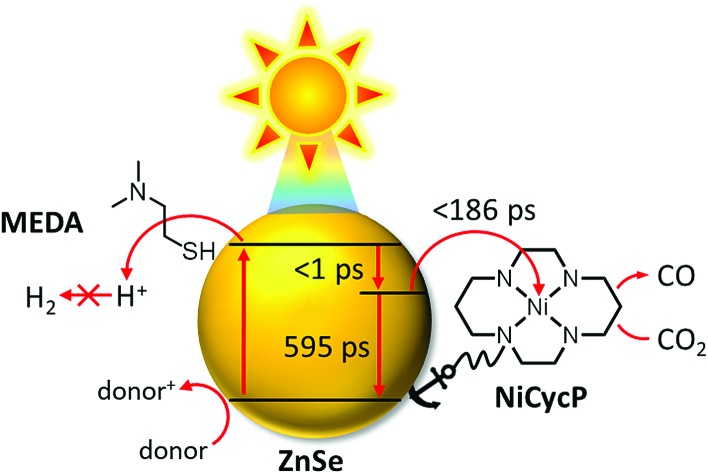
A robust precious metal-free photocatalyst system comprised of ligand-free ZnSe quantum dots and a phosphonic acid-functionalised Ni(cyclam) catalyst achieves efficient reduction of aqueous CO_2_ to CO.

## Introduction

Artificial photosynthesis allows for the storage of solar energy through the conversion of water and carbon dioxide into chemical fuels and represents a promising strategy to overcome the global dependence on fossil energy sources.^1^ Economic viability and scalability of this approach, however, greatly benefit from the development of environmentally benign photocatalysts consisting of inexpensive and abundant materials that operate in water. Hybrid photocatalysts have the potential to achieve this aim, because they combine the selectivity of molecular electrocatalysts with the photophysics of nanoparticulate photosensitisers.^2^ Ultra-fast electron-transfer kinetics often observed with quantum dots (QDs)^3^ are favourable for the overall efficiency, because productive electron transfer from the photosensitiser to the catalyst competes with charge recombination.^4^ Immobilising molecular catalysts on a semiconductor surface using suitable anchoring groups^5^ enables efficient charge transfer from the semiconductor to the catalyst.^6^

A growing number of earth-abundant molecular electrocatalysts capable of selective CO_2_ reduction in water, both in homogeneous phase^7^ and immobilised on electrodes^8^ have recently been reported. However, photocatalytic CO_2_ reduction is rarely achieved in aqueous solution without using precious metals.^9^ The potentials required to drive CO_2_ reduction at these catalysts are often very negative, resulting in competing proton reduction due to faster kinetics, and thus an overall low selectivity. Moreover, the necessary driving force is typically supplied by expensive light-absorbers such as Ir(ppy)_3_, Ru(bpy)_3_^2+^ or GaP.^10^ Entirely precious metal-free photocatalyst systems are scarce and only a few Fe^11^ and Co^12^ complexes have been reported to efficiently reduce CO_2_ when combined with inexpensive photosensitisers, but activity was only observed in organic solvents. Aqueous CO_2_ reduction using nickel terpyridine catalysts on CdS QDs has been reported with an efficiency of 0.28% (EQE_CO_) and a high CO selectivity of 90%.9*a* However, this system was limited by the durability of the catalyst, which started to degrade after 8 h of illumination. An Fe porphyrin-based system driven by an organic dye achieved a TON_CO_ of 120 and a selectivity of 95%, but was suffering from dye instability.9*d*

CO is one of the most versatile CO_2_ reduction products with numerous synthetic applications in industry including the synthesis of liquid fuels.^13^ Ni(cyclam)^2+^ is a non-precious electrocatalyst with high stability and activity for selective conversion of CO_2_ to CO.7d,e Photocatalytic reduction of aqueous CO_2_ has been achieved by combining Ni(cyclam)^2+^ and its derivatives with various photosensitisers.^14^ Nonetheless, these previous systems showed either good activity of up to 38 TON_CO_ with a poor selectivity of <10% or *vice versa* (TON_CO_ 2, CO selectivity 94%)14a,e despite using expensive photosensitisers such as Ru(bpy)_3_^2+^. Moreover, the observed selectivity for CO_2_ reduction over H_2_ evolution was often much lower than what is achieved in electrocatalytic CO_2_ reduction at Ni(cyclam)^2+^ and the activity and longevity was far below that of photocatalysts based on precious-metal electrocatalysts.^15^

**Fig. 1:**
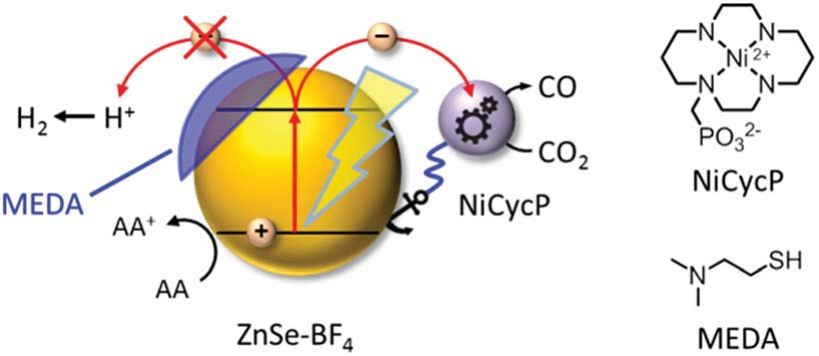
Schematic representation of the photocatalyst system developed in this work: ligand-free ZnSe QDs (ZnSe–BF_4_) combined with a molecular electrocatalyst, NiCycP, for aqueous CO_2_ reduction to CO. H_2_ evolution can be partially suppressed by a surface inhibitor (MEDA). AA: ascorbic acid.

Here, we present a novel hybrid photocatalyst system entirely free of precious metals (Fig. 1). The combination of inexpensive ZnSe QDs as a visible-light photosensitiser with a functionalised Ni(cyclam) electrocatalyst enables efficient CO_2_ reduction in aqueous solution with activities and longevities on par with precious metal-based systems by exploiting partial surface capping to enhance activity and selectivity. We use transient absorption spectroscopy to demonstrate that electrons are transferred from the photosensitiser to the immobilised catalyst on the ps-time scale, enabling fast catalyst turnover.

## Results and discussion

### Quantum dot synthesis

In line with ongoing efforts to replace scarce and expensive photosensitisers with inexpensive and benign materials,^16^ we first sought to identify a non-precious semiconductor material with a suitable conduction band (CB) edge to allow electron transfer to Ni(cyclam)^2+^. ZnSe is a stable semiconductor with a direct band gap of 2.7 eV,^17^ which enables absorption of near-UV and visible light, unlike ZnS which requires excitation by UV light.^18^ Additionally, electron mobility in ZnSe (610 cm^2^ V^–1^ s^–1^)^19^ is significantly higher than in its sulfide analogues ZnS (200 cm^2^ V^–1^ s^–1^) and CdS (385 cm^2^ V^–1^ s^–1^).^20^ Despite these favourable properties, ZnSe has received little attention for solar fuels generation. ZnSe-based photocathodes were recently reported for H_2_ evolution,^21^ but unlike their cadmium analogues CdS^22^ and CdSe,^23^ ZnSe QDs have not been used in photocatalytic reduction of aqueous protons or CO_2_. At pH 5.5, the ZnSe CB is located at approximately –1.4 V *vs.* NHE,21*a**i.e.* 400 mV more negative than the onset potential for electrocatalytic CO_2_ reduction at Ni(cyclam)Cl_2_ of approximately –1.0 V *vs.* NHE at pH 5.5 (*E*^0^′_CO_2_/CO_ = –0.43 V *vs.* NHE).14*b* While the energetics suggest that electron transfer from the ZnSe CB to Ni(cyclam)^2+^ is thermodynamically feasible, the electron transfer kinetics are crucial to efficiently drive CO_2_ reduction at Ni(cyclam)^2+^. Electron transfer from the photosensitiser to the co-catalyst must be faster than charge recombination, and CO_2_ reduction additionally competes with H_2_ evolution.

We have previously demonstrated that capping ligands, commonly employed to control QD growth and stability, can significantly impact their photocatalytic activity.^24^ Ligand removal exposes vacant surface sites^25^ that are needed for the controlled immobilisation of anchor-functionalised molecules.9*a* The ligand-free QDs used here enable us to study the effect of first adding defined amounts of a CO_2_ reduction catalyst, followed by blocking the remaining particle surface with capping ligands to suppress competing H_2_ evolution.

**Fig. 2:**
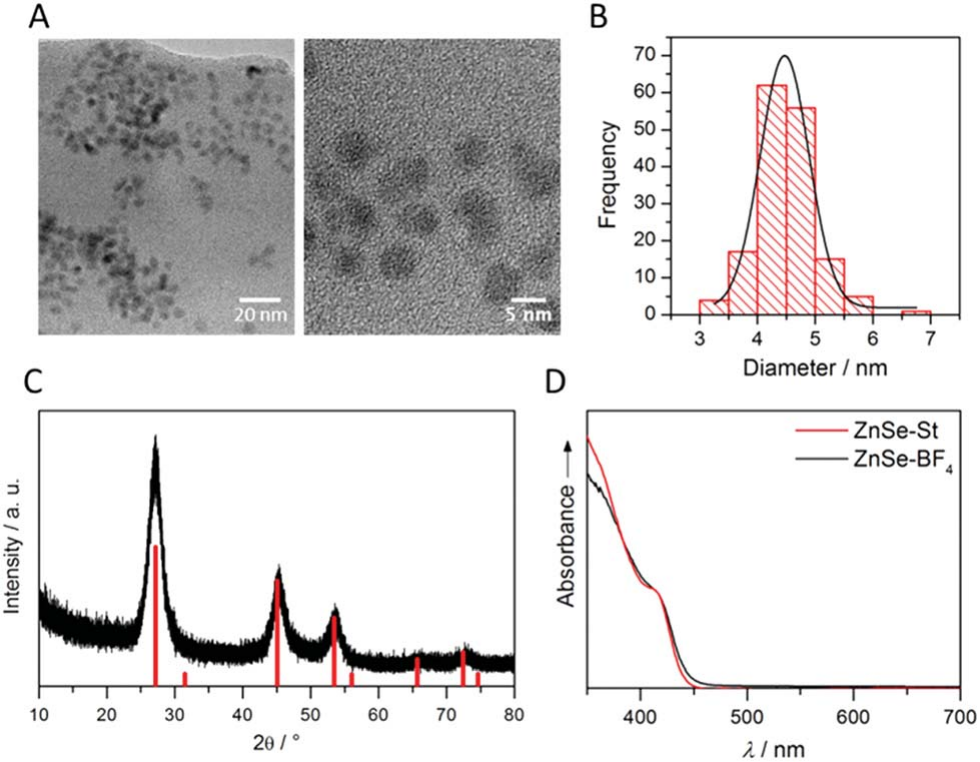
Characterisation of ligand-free ZnSe QDs (ZnSe–BF_4_): (A) transmission electron micrographs with (B) corresponding particle size distribution. (C) Powder X-ray diffractogram overlaid with cubic zinc blende ZnSe reference (PDF 01-071-5978); (D) UV-vis spectrum compared to ZnSe–St.

ZnSe QDs were prepared using a modified literature procedure based on heating zinc stearate and selenium in octadecene to 300 °C.^26^ A growth period of 130 min yielded individual, near-spherical stearate-capped ZnSe nanocrystals (ZnSe–St). An average diameter of 4.55 ± 0.62 nm was determined from transmission electron microscopy (TEM, Fig. S1A and B†) in good agreement with a diameter of 5.40 ± 0.93 nm determined from powder X-ray diffraction (XRD, Fig. S1C†) using the Scherrer equation.^27^ Ligand-free, charge stabilised ZnSe QDs (ZnSe–BF_4_) were obtained from ZnSe–St by reactive ligand stripping using Me_3_OBF_4_.^28^ ZnSe QDs show little change in the particle size, morphology and structure upon ligand stripping; the mean particle size was 4.50 ± 0.53 nm (from TEM; 5.02 ± 0.41 from XRD, Fig. 2A–C). The UV-vis spectrum features good visible-light response with a first excitonic absorption maximum at 417 nm (Fig. 2D). ATR-IR spectroscopy of dried ZnSe–BF_4_ particles shows the expected signatures of surface-adsorbed BF_4_^–^ (1010 cm^–1^) and DMF (1089, 1375 and 1649 cm^–1^), additional bands at 730 and 1537 cm^–1^ suggest a small amount of residual stearate on the particle surface (Fig. S2†). X-ray photoelectron spectroscopy (XPS) shows a 0.8 eV shift of the O_1s_, Zn_2p_ and Se_3d_ binding energies to more positive for ZnSe–BF_4_ particles compared to ZnSe–St, suggesting partial oxidation of the QD surface upon ligand stripping (Fig. S3†).

### Photocatalytic CO_2_ reduction

A hybrid photocatalyst system was subsequently assembled, using ZnSe–BF_4_ as the light absorber to drive aqueous CO_2_ reduction at the molecular co-catalyst NiCycP,14*b* a functionalised derivative of Ni(cyclam)Cl_2_ bearing a phosphonic acid anchoring group (Fig. 1). Adding NiCycP to an aqueous suspension of ZnSe–BF_4_ resulted in attachment of the co-catalyst to the particle surface. ATR-IR spectra of ZnSe–BF_4_ isolated and washed after NiCycP immobilisation showed the appearance of additional bands at 1048, 965 and 580 cm^–1^ corresponding to attached NiCycP (Fig. S4†). Ion-coupled plasma optical emission spectroscopy (ICP-OES) confirmed that 7.8 ± 0.5% of the added NiCycP was immobilised (corresponding to 2 catalyst molecules per QD), whereas the non-phosphonated analogue Ni(cyclam)Cl_2_ showed a lower attachment of only 2.9 ± 0.2% (Table S1†).

**Fig. 3:**
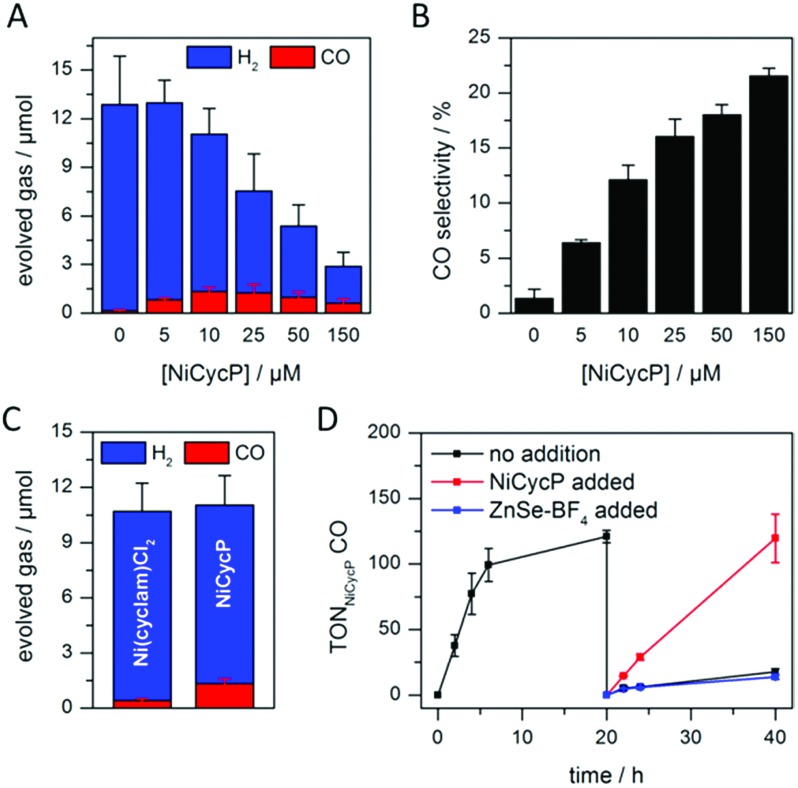
Photocatalytic reduction of aqueous CO_2_ in the presence of ZnSe–BF_4_/NiCycP: (A and B) effect of varying co-catalyst loadings on product distribution and selectivity (4 h irradiation). (C) Performance depending on the employed co-catalyst (4 h irradiation). (D) Long-term activity of ZnSe–BF_4_/NiCycP (sample purged with CO_2_ after 20 h and 0.5 μM ZnSe–BF_4_, 10 μM NiCycP or nothing added). Conditions: AM 1.5G, *λ* > 400 nm, 100 mW cm^–2^, 0.5 μM ZnSe–BF_4_, 10 μM NiCycP (for C and D), 0.1 M AA, pH 5.5, CO_2_, 25 °C (unless otherwise stated).

The photocatalytic performance of this hybrid assembly was studied in CO_2_-saturated water under UV-filtered simulated solar light irradiation (AM 1.5G, *λ* > 400 nm, 100 mW cm^–2^) using ascorbic acid (AA, 0.1 M, pH 5.5) as sacrificial electron donor. In the absence of a co-catalyst, ZnSe–BF_4_ photocatalytically reduces protons to H_2_, indicating that electron transfer to aqueous protons can, indeed, compete with charge recombination. Fig. 3A and B show that adding NiCycP to ZnSe–BF_4_ promotes the generation of CO at the expense of H_2_ evolution (Table S2†). This observation suggests that H_2_ evolution at the particle surface and CO_2_ reduction at the co-catalyst directly compete for CB electrons, thus proving electron transfer to NiCycP to be kinetically feasible. The importance of fast electron transfer from ZnSe to the co-catalyst was further demonstrated by employing the freely diffusing co-catalyst Ni(cyclam)Cl_2_ instead of anchored NiCycP. The CO_2_ reduction activity was over three times lower (Fig. 3C, Table S2†), even though Ni(cyclam)Cl_2_ shows a higher level of activity as a homogenous electrocatalyst and has a similar onset potential for CO_2_ reduction.14*b* We have previously reported that co-immobilisation of NiCycP and a Ru-dye on a solid support accelerates electron transfer from the dye to the catalyst compared to a diffusional homogeneous system.14b,^29^

Under optimised conditions (10 μM NiCycP), ZnSe–BF_4_/NiCycP achieved a Ni-based TON_CO_ of up to 121 ± 6 and 8.0 ± 0.9% CO selectivity after 20 h. Higher catalyst loadings (150 μM NiCycP, Fig. 3B) led to a higher CO selectivity of up to 21.5 ± 1.1%, but resulted in a lower TON_CO_ (Table S2†). The photocatalyst system is still active after 20 h irradiation, but the rate of CO production is lowered while H_2_ generation remains largely unchanged. Adding fresh ZnSe–BF_4_ had little effect on the CO production, whereas adding fresh NiCycP restored the initial activity and selectivity (Fig. 3D, S5†), indicating that ZnSe–BF_4_ remains intact while the molecular co-catalyst undergoes deactivation over time, presumably due to CO poisoning,^30^ or decomposition. The stability of the particles was further corroborated by UV-vis spectra collected after irradiation (Fig. S6†). Increased scattering implies some particle agglomeration, however the absorption onset of the ZnSe–BF_4_ QDs remains unchanged compared to a sample that was stirred in the dark or the stock solution. TEM images of the particles after photocatalysis confirmed the formation of aggregates which retained a nanocrystalline morphology (Fig. S7†). Only ^13^CO was generated when photocatalysis was performed under ^13^CO_2_, confirming CO_2_ as the sole carbon source (Fig. S8†). Control experiments in the absence of NiCycP yielded only traces of CO; no CO was formed without ZnSe or in the dark (Table S3†). Experiments in the absence of AA gave only negligible amounts of CO and H_2_, suggesting that particle self-oxidation is not a major process as previously seen for CdSe.^31^

### Enhanced selectivity with partial ligand capping

**Fig. 4:**
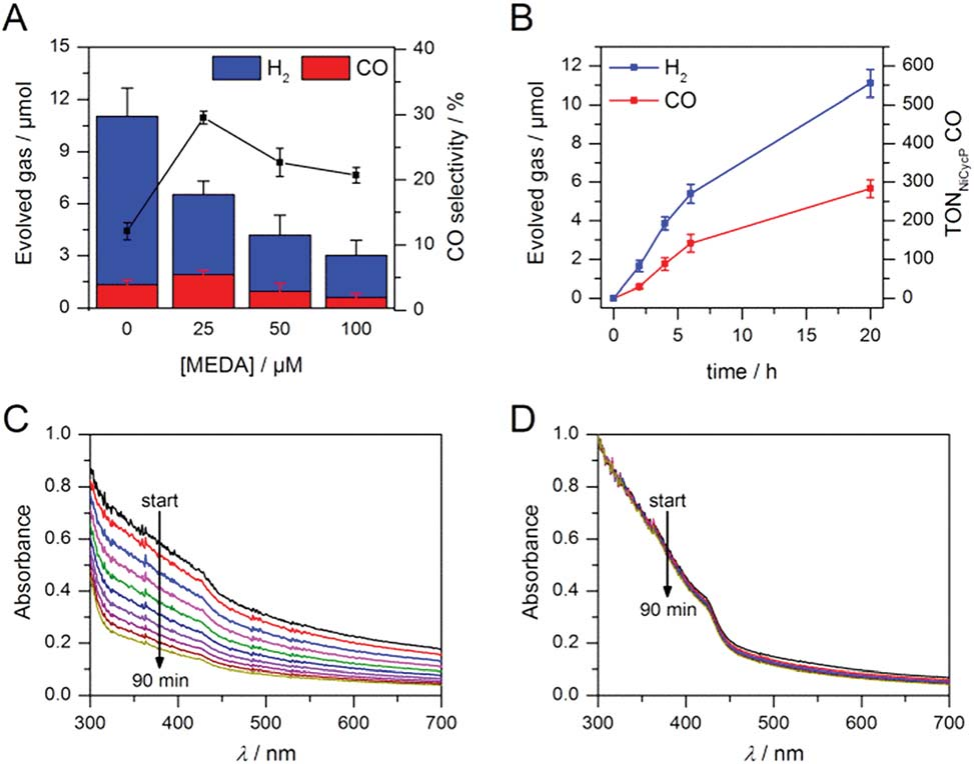
Enhanced CO_2_ photoreduction with ZnSe–BF_4_/NiCycP in the presence of MEDA: (A) changes in product selectivity with different MEDA concentrations (4 h irradiation). (B) Long-term activity of ZnSe–BF_4_/NiCycP/MEDA (25 μM MEDA). Conditions: AM 1.5G, *λ* > 400 nm, 100 mW cm^–2^, 0.5 μM ZnSe–BF_4_, 10 μM NiCycP, 0.1 M AA, pH 5.5, CO_2_, 25 °C. (C and D) Change of the UV-visible spectrum of aqueous ZnSe–BF_4_ over 90 min in the absence (C) or presence (D) of added MEDA (0.5 μM ZnSe–BF_4_, 0.1 M AA, pH 5.5, 25 °C, with or without 25 μM MEDA, no irradiation).

Having demonstrated how co-catalyst immobilisation can accelerate the desired electron transfer kinetics, we sought to further enhance the product selectivity by decelerating H_2_ evolution from the particle surface. Our previous work has shown that capping ligands can inhibit photocatalytic H_2_ evolution at CdS quantum dots.^24^ Here, we exploit this effect to control the product selectivity in CO_2_ reduction. We studied the influence of adding 2-(dimethylamino)ethanethiol (MEDA) on the photocatalytic activity of ZnSe–BF_4_/NiCycP at a catalyst loading (10 μM NiCycP) where the CO selectivity was low (8.0 ± 0.9%). CO_2_ photoreduction was performed in the presence of 25, 50 or 100 μM MEDA to partially passivate the particle surface and thereby lower H_2_ evolution activity. Fig. 4A confirms that adding MEDA suppresses proton reduction at ZnSe–BF_4_/NiCycP already at low concentrations, while CO evolution was even enhanced at low MEDA concentrations and only suppressed at higher loadings. At MEDA concentrations >150 μM, overall photocatalytic activity was negligible, demonstrating that fully ligand-capped QDs are inactive. At low concentrations (25 μM), MEDA therefore increases the selectivity for CO_2_ reduction from 8.0 ± 0.9% to 33.8 ± 1.7% by selectively suppressing the competing H_2_ evolution. The generated syngas had an ideal H_2_ : CO ratio of 2 : 1 as required for the industrial production of liquid fuels such as methanol and hydrocarbons.13*b* In addition, improved CO production was observed, enabling NiCycP to reach an unprecedented TON_CO_ of 283 ± 23 after 20 h (Fig. 4B, Table S4†), presumably because more excited charges are available for the molecular catalyst.

ICP-OES confirmed that at the optimum MEDA concentration (25 μM), the QDs are only partially covered (23 equiv. MEDA adsorbed per QD with approx. 100 surface Zn atoms per QD). At higher MEDA concentrations where full coverage is expected, CO production was inhibited, suggesting that the NiCycP catalyst is displaced from the QD surface. UV-vis spectroscopy showed that MEDA, in addition to enhancing the product selectivity, improves ZnSe–BF_4_ particle stability in solution (Fig. 4C and D). In the absence of MEDA, a decrease in absorbance and strong scattering is observed over time, whereas MEDA-containing samples remain unchanged over the course of 90 min. Zeta potential measurements confirmed that adding MEDA results in an increase in positive particle charge (Table S5†), presumably due to protonation of the tertiary amine at the employed conditions (pH 5.5). This positive charge can stabilise the colloidal suspension by coulombic repulsion. The presence of MEDA did not have a significant impact on surface oxidation of the ZnSe–BF_4_ particles as confirmed by XPS measurements (Fig. S3†). We expect suppressed H_2_ evolution to result from MEDA blocking Zn sites on the particle surface.

For ZnSe–BF_4_/NiCycP/MEDA, the average external quantum efficiency for CO production (EQE_CO_) under 400 nm monochromatic light was 3.4 ± 0.3% (1 mW cm^–2^, average taken over 6 h; Table S6†); H_2_ evolution showed an EQE_H_2__ of 4.2 ± 0.2%. This performance considerably exceeds previous results on photocatalytic CO_2_ reduction at Ni(cyclam)^2+^ and derivatives (Table 1).^14^ The highest photocatalytic activity has been previously reported for Ni(cyclam)^2+^ embedded in a Cu–azurin protein scaffold, achieving a TON_CO_ of 38 and <10% selectivity with a Ru(bpy)_3_^2+^ sensitiser.14*a* 94% selectivity and a TON_CO_ of 2.2 have been reported for a dinuclear Ni(cyclam)-derivative sensitised by Ru(bpy)_3_^2+^.14*e* An EQE_CO_ of 0.14% was reported for a Ni(cyclam)^2+^/Ru(bpy)_3_^2+^ combination in a biphasic H_2_O/scCO_2_ mixture.14*d* An analogous Co(cyclam)^3+^ catalyst immobilised on Ru(bpy)_3_^2+^-sensitised TiO_2_ was reported to achieve a TON_CO_ of 35 and <20% selectivity.9*c* The best performing synthetic non-precious photocatalyst system in water, an Fe porphyrin sensitised by a homogeneous organic dye, achieved a TON_CO_ of 120 and 95% selectivity after 94 h irradiation. However, the organic dye degraded over time with more dye consumed than CO produced (TON_sensitiser_ = 0.6),9*d* whereas in our system the ZnSe QDs are stable and achieve a TON_QD_ > 5000 mol CO mol^–1^ QD. A CdS/Ni(terpyridine) hybrid achieved a TON_CO_ of 20 and an EQE_CO_ of 0.28%.9*a* TONs in water comparable to the present work have only been reported for precious metal-based catalysts. A Ru–Re vesicle system achieved a TON_CO_ of 190 and showed an excellent 98% selectivity in aqueous solution at pH 6.5–7.1 (no EQE reported).15*f* A homogeneous Ru–Re dyad achieved a TON_CO_ of 130, 81% selectivity and a remarkable EQE_CO_ of 13% in pH 9.8 aqueous solution.15*c* In mixed organic/aqueous solution, a ReP catalyst immobilised on dye-sensitised TiO_2_ showed a TON_CO_ of 86 with tuneable selectivity up to 78% selectivity.^33^ Considerably higher TONs have been achieved using a carbon monoxide dehydrogenase (CODH) enzyme immobilised on Ru-sensitised TiO_2_,^32^ or on CdS nanorods,9*b* but these systems showed low quantum yields and the enzymes are extremely fragile as well as expensive and difficult to isolate and purify. Higher performances have been achieved with precious-metal-based catalysts for CO_2_ photoreduction to formic acid.5a,5b Anchoring dinuclear Ru complexes on Ag-loaded TaON allowed for a TON up to 750 and 85% selectivity in water (0.47% EQE).15*b* Replacing TaON with graphitic carbon nitride showed enhanced performance (TON > 2000, up to 98% selectivity, EQE 0.2%) in water,15*a* and a remarkable TON > 33 000 when organic solvents were used instead.15*d*

**Table 1:** Literature comparison of different catalysts for visible-light driven reduction of CO_2_ to CO in aqueous solution

Photocatalyst system		TON_CO_ [mol_CO_/mol_cat_]	Selectivity^*a*^ [%]	EQE_CO_ [%]	Ref.
Light absorber	Catalyst				
Ru(bpy)_3_^2+^	Ni(cyclam)@Cu–azurin	38	<10	n.r.^*b*^	14*a*
Ru(bpy)_3_^2+^	[Ni(cyclam)]_2_^4+^	2.2	94	n.r.	14*e*
Ru(bpy)_3_^2+^	Ni(cyclam)^2+^	2.1	87	0.14	14*d*
Ru(bpy)_3_^2+^	Ni(cyclam)^2+^	0.1	13	n.r.	14*g*
Ru(bpy)_3_^2+^	Ni(cyclam)^2+^	n.r.	80	0.06	14*f*
RuP/ZrO_2_	NiCycP	4.8	19	n.r.	14*b*
Ru–cyclam–Ni dyad		5.2	71	n.r.	14*c*
Ru(bpy)_3_^2+^/TiO_2_	Co(cyclam)^3+^	35	<20	n.r	9*c*
Ru(dmb)_2_–Re(CO)_3_Cl^2+^ dyad		130	81	13	15*c*
[Ru(dtb)(bpy)_2_]^2+^	Re(dtb)(CO)_3_Cl	190	98	n.r.	15*f*
CdS	CODH enzyme	22 500	n.r.	0.01	9*b*
RuP/TiO_2_	CODH enzyme	2 100	n.r.	n.r.	32
CdS	Ni(terpyS)_2_^2+^	20	>90	0.28	9*a*
Purpurin	Fe-*p*-TMA	120	95	n.r.	9*d*
ZnSe–BF_4_/MEDA	NiCycP	283 ± 23	34 ± 2	3.4 ± 0.3	This work

^*a*^Selectivity = 100% × *n*_CO_/(*n*_CO_ + *n*_H_2__).

^*b*^n.r. = not reported.

### Ultrafast transient absorption (TA) spectroscopy

**Fig. 5:**
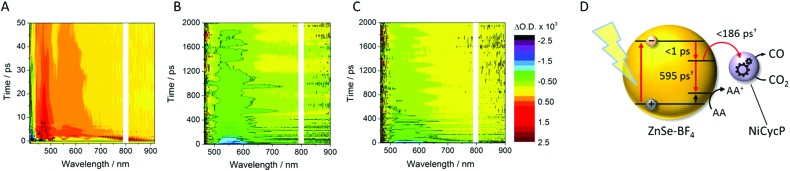
TA spectroscopy of the ZnSe–BF_4_/NiCycP/MEDA photocatalyst under different conditions: band gap excitation of ZnSe–BF_4_/MEDA (A) in the absence of AA produces a ground state bleach and a positive feature (hole), (B) in the presence of AA produces a long-lived red-shifted bleach (trapped electrons), and (C) in the presence of NiCycP and AA accelerates recovery of the trap state bleach (conditions: 0.5 μM aqueous ZnSe–BF_4_, pH 6.5, with or without 0.1 M AA, with or without 25 μM MEDA, with or without 10 μM NiCycP under Ar at room temp.; 400 nm excitation, 10 nJ; 450–900 nm probe). (D) Kinetics of the observed trapped electrons derived from exponential fitting († amplitude-weighted average lifetime, see Fig. S9–S11 and Table S7† for details).

TA spectroscopy can offer important insights into the factors controlling photocatalyst activity through the analysis of charge carrier dynamics. Here, we use TA spectroscopy to elucidate the electron transfer kinetics in ZnSe–BF_4_/NiCycP to unravel the mechanism behind its high photocatalytic performance (Fig. 5). The TA spectrum of ZnSe–BF_4_/MEDA in water was measured following 400 nm excitation (Fig. 5A, see Fig. S9–S11† for detailed description). The spectra show a ground state bleach at 425 nm, which decays very rapidly (*τ*_1_ = 0.9 ± 0.2 ps, *τ*_2_ = 35 ± 15 ps; see Fig. S10, Table S7† for full fitting parameters). We also observe photoinduced absorptions at 470 nm and *ca.* 590 nm within 2 ps of excitation. The absorptions at 470 nm and 590 nm have a significantly longer lifetime (470 nm: *τ*_1_ = 25 ± 4 ps, *τ*_2_ = 409 ± 106 ps; 590 nm: *τ*_1_ = 21 ± 6 ps, *τ*_2_ = 475 ± 72 ps) than the ground state bleach. The bleach feature in such systems typically arises due to state-filling by excited electrons in conduction band states,^34^ while the broad positive signal has been ascribed to trapped charge carriers.^35^ The lifetimes of the bleach and photoinduced absorption do not match, indicating that different relaxation pathways are available. This observation is in line with past studies which show the presence of a range of mid-gap states with similar ZnSe materials^36^ and our expectation of additional surface states that are likely to be present as a consequence of ligand stripping. In the presence of AA, the positive features at 470 and 590 nm are removed (Fig. 5B). This supports their assignment to trapped charge carriers, specifically holes, and is consistent with a reductive quenching mechanism. We now observe a broad bleach centred at 550 nm. Previous TA studies of ZnSe report the occupation of defect states below the CB edge by photoelectrons,35a,^37^ with the state filling leading to an increase in transmission at energies below the band gap. Therefore, we assign the broad bleach in Fig. 5B to trapped photoelectrons.

**Fig. 6:**
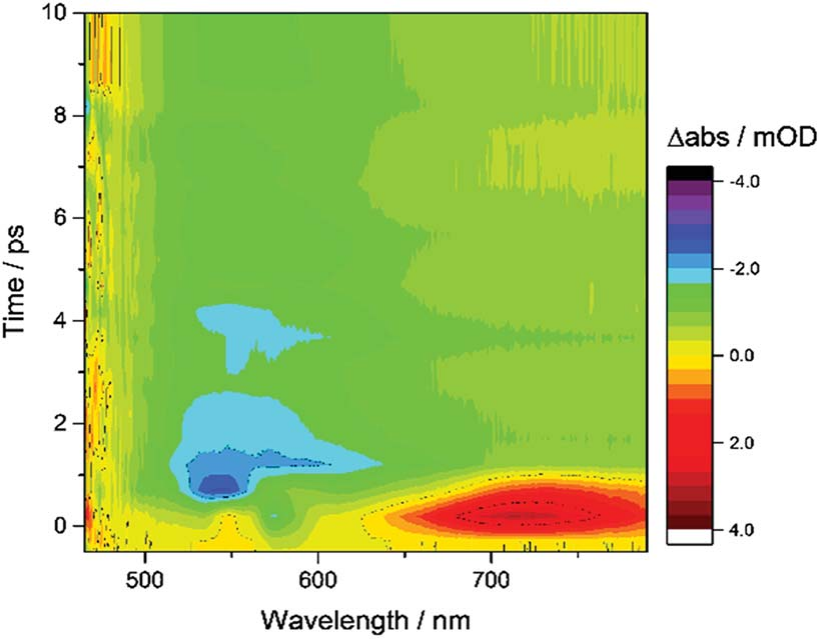
TA spectroscopy of ZnSe–BF_4_/MEDA (0.5 μM ZnSe–BF_4_, 25 μM MEDA) in aqueous solution in the presence of AA (0.1 M, pH 6.5, Ar, room temperature), showing the first 10 ps following excitation (400 nm).

Fig. 6 shows the first 10 ps following excitation of ZnSe–BF_4_/MEDA in the presence of AA. Here we observe loss of the initially generated photoinduced absorptions and formation of the trapped photoelectron signal within *ca*. 1 ps of excitation, indicating that both hole scavenging and electron trapping can occur on the ultrafast timescales. Hole scavenging rates on the order of 10^12^ s^–1^ and greater have been previously reported for II–VI nanocrystals elsewhere^38^ and recent studies have also observed sub-ps trapping in ZnSe, potentially of hot electrons37*a* making accurate measurement of the kinetics of these processes beyond the resolution of our spectrometer (400 fs).

In the presence of AA and NiCycP (Fig. 5C), recovery of the bleach centred at 550 nm assigned to trapped electrons is clearly accelerated, suggesting electron transfer from the trap to the molecular catalyst (Fig. 5D), and further confirming the assignment of the negative feature to trapped photoelectrons. Data shown in Fig. 5 was collected under Ar and the electron transfer will correspond to the initial reduction of Ni^II^CycP to Ni^I^CycP. CO_2_ reduction requires the accumulation of an additional electron on the catalyst making it important that the initially reduced state is sufficiently long-lived. In the ps-TA experiments carried out, we are unable to directly probe NiCycP, a complex that only absorbs very weakly in the visible region,14*b* however Fig. 5 indicates that under the conditions employed in the catalysis and TA studies, AA efficiently scavenges ZnSe photoholes which would minimise back electron transfer from Ni^I^CycP to ZnSe. Fitting of the bleach recovery is complicated and requires a minimum of a triexponential function with a small residual signal (17% for QD/AA, 10% for QD/AA/NiCycP) remaining at the longest timescale we can study (3 ns, Fig. S11, Table S7†). In similar systems where complex multi-exponential decay kinetics have been observed in the study of charge transfer from QDs, the amplitude-weighted averaged lifetime has been reported,^39^ which can provide a simple measure of the average lifetime. In the presence of NiCycP, a large decrease in the amplitude-weighted average lifetime from 595 to 186 ps is observed, giving an approximate electron transfer lifetime to the catalyst.^39^ Electron transfer between ZnSe–BF_4_ and NiCycP is thus much faster than in previous work using NiCycP and a Ru dye14*b* (transfer lifetime of 130 μs) or Ni(cyclamCO_2_H)^2+^ on TiO_2_ (*t*_50%_ = 1.2 ms).^40^ Faster charge transfer times have been reported for other systems, *e.g.* from CdSe QDs to an immobilised Re complex (2.3 ps),6*a* from N–Ta_2_O_5_ to an anchored Ru complex (12 ps)6*b* and from CuInS_2_ QDs to an adsorbed Fe porphyrin (<200 fs).^41^ The rapid electron transfer coupled to the observed ultrafast formation (approximately 1 ps) of longer-lived trapped electrons gives an explanation for the high performance of the presented system.

## Conclusions

In summary, we have established ZnSe QDs as an inexpensive, Cd-free and stable photosensitiser for artificial photosynthesis. A hybrid photocatalyst consisting of ZnSe–BF_4_ QDs and immobilised NiCycP reduces aqueous CO_2_ to CO with high TONs under visible-light irradiation without the use of precious metals. Anchor-free Ni(cyclam)^2+^ shows a substantially lower affinity for the QD surface, which is consistent with a much lower photocatalytic activity. A detailed study of the electron transfer kinetics using TA spectroscopy revealed ultra-fast trapping of conduction band electrons, followed by fast and efficient electron transfer from these long-lived trap states to the immobilised NiCycP on the ps timescale. These rapid electron transfer dynamics are thus key to the high performance of the ZnSe–NiCycP photocatalyst system. We further demonstrate that the selectivity for CO_2_ reduction could be increased by suppressing H_2_ evolution and enhancing CO generation through blockage of available QD surface sites by a capping ligand. The optimised ZnSe–BF_4_/NiCycP/MEDA photocatalyst achieved a performance comparable to the best precious metal-based photocatalysts with a TON_CO_ > 280 and 3.4% EQE_CO_, producing CO and H_2_ in a 1 : 2 ratio, *i.e.* solar syngas.

## Experimental section

### Materials

All chemicals were obtained from commercial sources and used as received. l-Ascorbic acid (99%), *N*,*N*-dimethylformamide (DMF, 99.8%), octadecene (90% techn.), selenium powder (99%), trimethyloxonium tetrafluoroborate (96%) and zinc stearate (purum) were purchased from Sigma-Aldrich. 2-Dimethylaminoethanethiol hydrochloride (MEDA, 95%) was purchased from Acros Organics, 1-butanol (99%) was purchased from Alfa Aesar. All aqueous experimental solutions were prepared with distilled water and all aqueous analytical samples were prepared with ultrapure water (DI water; Milli-Q®, 18.2 MΩ cm). ^13^CO_2_ (>99 atom% ^13^C) was purchased from Sigma Aldrich. Ni(cyclam)Cl_2_,7*e* and NiCycP14*b* were prepared by literature procedures.

### ZnSe–St

Stearate capped ZnSe-QDs were prepared by a modified literature procedure^26^ as follows: zinc stearate (1 mmol), Se powder (1 mmol) and 65 mL octadecene were added to a 250 mL three-necked flask and degassed for 1.5 h at 50 °C. The reaction was triggered by raising the temperature to 300 °C under a N_2_ atmosphere, resulting in an initially colourless reaction mixture that turned progressively yellow. To monitor particle growth, aliquots (100 μL) were taken periodically, diluted with CHCl_3_ to 1 mL total volume, filtered with a syringe filter (Merck Millex-GN, 0.20 μm nylon membrane) and analysed by UV-vis spectroscopy. After 2 h 10 min (counted from the time when the temperature was raised above 50 °C) the reaction was stopped by removing the heating mantle and blowing N_2_ into the flask. The particles were precipitated using an acetone/methanol mixture (20 : 80), followed by centrifugation (7000 rpm, 10 min). The residue was washed with methanol (twice) and butanol and re-dispersed in CHCl_3_.

### ZnSe–BF_4_

Ligand-free ZnSe QDs were prepared by reactive ligand removal using a modified literature procedure for CdS stripping.^24^,^28^ A ZnSe–St solution in CHCl_3_ was dried *in vacuo*. Under a N_2_ atmosphere, the residue was re-dispersed in a mixture of anhydrous CHCl_3_ (3 mL) and anhydrous DMF (0.2 mL). Aliquots of stripping agent (Me_3_OBF_4_, 1.0 M in acetonitrile, typically 2–3 mL) were added slowly until the particles precipitated. The resulting ligand-free particles were centrifuged (7000 rpm, 10 min), dried in air for 1 min, and re-dispersed in DMF (2–3 mL). The resulting slightly cloudy solution of ZnSe–BF_4_ in DMF was further purified by centrifugation (7000 rpm, 10 min) to give a black precipitate, a clear yellow solution and a cloudy white phase on top. The black precipitate and white phase were removed and the clear yellow solution was used for characterisation and photocatalytic experiments. Thus-prepared ZnSe–BF_4_ can be handled in air for hours without decomposition but will gradually degrade over several days in air. To prevent degradation, the ZnSe–BF_4_ solution was degassed by 4 freeze–pump–thaw cycles and stored under N_2_ in the dark at 4 °C. The mean particle size was determined from TEM images and was found in good agreement with the average diameter determined from applying the Scherrer equation to the XRD pattern (see ESI for details†).^27^ To calculate the QD concentration in the stock solution, the Zn^2+^ concentration determined by ICP-OES, was divided by the number of Zn atoms per QD based on the mean particle diameter and the bulk density of ZnSe.

### Photocatalytic CO_2_ reduction

A ZnSe–BF_4_ stock solution (144.7 μM in DMF, 6.90 μL) and a co-catalyst solution (2.0 mM NiCycP or Ni(cyclam)Cl_2_ in water, typically 10 μL) were added to a Pyrex glass photoreactor (Chromacol 10-SV, Fisher Scientific) containing a magnetic stirrer bar. The mixture was diluted with ascorbic acid (AA, 0.1 M in water, pH adjusted to 6.5 with NaOH) to a total solution volume of 2 mL. The photoreactor was then sealed with a rubber septum and purged with CO_2_ (containing 2% CH_4_ as internal standard) for 10 min in the dark; the solution pH decreased from 6.5 to 5.5 after purging due to saturation with CO_2_. The photoreactor was then placed in a water bath maintained at 25 °C, stirred and irradiated by a solar light simulator (Newport Oriel, 100 mW cm^–2^) equipped with an air mass 1.5 global (AM 1.5G) filter. IR irradiation was filtered with a water filter (10 cm path length) and UV irradiation with a 400 nm cut-off filter (UQG). Product distribution was quantified through periodic headspace gas analysis (50 μL) by gas chromatography (see ESI for details†). For isotopic labelling, photocatalysis experiments were performed as described above, but using ^13^CO_2_ as the headspace gas (see ESI for details†). After 15 h, the photoreactor headspace was transferred to an evacuated gas IR cell (SpecAc, 10 cm path length, equipped with KBr windows) and a high-resolution gas-phase transmission spectrum was collected.

### Analysis of catalyst loading on QDs

Samples were prepared as described above for photocatalysis experiments, but scaled up by a factor of 13 for accurate determination of the Ni^2+^ concentration (0.5 μM QD-BF_4_, 10 μM catalyst in 26 mL 0.1 M aq. AA, pH 6.5). Samples were purged with CO_2_ for 10 min, stirred for 2 h in the dark before the particles were separated by centrifugation (6500 rpm, 30 min). The supernatant was discarded and the precipitate was dissolved in 1 mL concentrated HNO_3_ (trace metal analysis grade), diluted with water (1 : 200 for Zn^2+^, 1 : 10 for Ni^2+^) and analysed by ICP-OES. The catalyst loading was calculated from the relative Ni^2+^ and Zn^2+^ concentrations in the precipitate. To study immobilised NiCycP on ZnSe–BF_4_ by ATR-IR spectroscopy, one drop of QD stock solution was dried on a fluorine-doped tin oxide (FTO)-coated glass slide *in vacuo* and the FTO/ZnSe–BF_4_ was incubated with water or aqueous NiCycP (1.0 mM) for 2 h, carefully washed with water and dried *in vacuo* before collecting spectra.

### Transient absorption (TA) spectroscopy

Femtosecond TA spectroscopy was carried out using a PHAROS laser (Light Conversion, Ltd) operating at 10 kHz coupled to an ORPHEUS optical parametric amplifier (Light Conversion, Ltd) in tandem with a LYRA harmonic generator (Light Conversion, Ltd) to produce the desired wavelength for sample excitation. The pump beam intensity was adjusted with a neutral density filter so as to achieve approximately equal photon fluxes at different wavelengths. Typical pulse energies were on the order of 10 nJ. The pump wavelength was tuned to 400 nm. A portion of the PHAROS output was also split off to pump a sapphire crystal to generate a white light continuum for the probe beam, which provided for spectral observation in the region 450–900 nm. The probe beam was focused to a spot size of ∼100 μm diameter on the sample and was overlapped completely by the pump beam. Spectra were acquired with a HELIOS transient absorption spectrometer (Ultrafast Systems, LLC). The time resolution of the setup is *ca.* 400 fs. Measurements were performed by randomly stepping the optical delay line and averaging for 1 s at each delay time. 3 to 5 consecutive scans were collected and aggregated to produce each spectrum. Sample solutions were prepared in a similar fashion to those for photocatalysis experiments (0.5 μM QD, 10 μM NiCycP in 0.1 M aq. AA, pH 6.5) unless otherwise stated, purged with Ar and transferred to a 1 or 2 mm path length quartz cuvette under an inert atmosphere.

## Conflicts of interest

The authors declare no competing financial interests.

## Supplementary Material

Supplementary information
