# Supplementary material for: ZnSe quantum dots modified with a Ni(cyclam) catalyst for efficient visible-light driven CO2 reduction in water
Source: Chem Sci. 2018 Jan 24;9(9):2501–9. doi: 10.1039/c7sc04429a (PMC5911736; doi:10.1039/c7sc04429a)
Supplement: Supplementary file 1 [file SC-009-C7SC04429A-s001.pdf]

Electronic Supplementary Material (ESI) for Chemical Science.
This journal is © The Royal Society of Chemistry 2018

Supporting Information

ZnSe quantum dots modified with a Ni(cyclam) catalyst for efficient visible-light driven CO_2 reduction in water

Moritz F. Kuehnel[a†§], Constanting D. Sahm[a†], Gaia Neri[b], Jonathan R. Lee[b], Katherine L. Orchard[a], Alexander J. Cowan[b]* and Erwin Reisner[a]*

[a] Christian Doppler Laboratory for Sustainable SynGas Chemistry, Department of Chemistry, University of Cambridge, Lensfield Road, Cambridge CB2 1EW, UK

[b] Stephenson Institute for Renewable Energy, Department of Chemistry, The University of Liverpool, Crown Street, Liverpool L69 7ZD, UK

§ Present address: Department of Chemistry, Swansea University, College of Science, Singleton Park, Swansea SA2 8PP, UK

†These authors contributed equally.

*Corresponding author email addresses:
reisner@ch.cam.ac.uk
A.J.Cowan@liverpool.ac.uk

Experimental Section

Powder X-ray diffraction (XRD). XRD was conducted using an X'Pert PRO by PANalytical BV instrument using CuK_α irradiation. Particle sizes were determined from XRD data using the full width at half maximum (FWHM) of a given Bragg reflection according to equation 1.[1]

$$d = \frac{4}{3} \times \frac{0.9\,\lambda}{\omega \cos\theta} \qquad (1)$$

Where d is the crystallite size, λ is the X-ray irradiation wavelength, θ is the angle of the considered Bragg reflection and ω is the FWHM on a 2θ scale. In this study, CuK_α irradiation was used with a weighted average of λ = 1.5418 Å. (CuK_α(1) 1.54059 Å and CuK_α(2) 1.54443 Å). In order to determine the position and FWHM of each reflection, a baseline-corrected Gauss fit of the XRD diffractogram was performed. The mean crystallite size was calculated from averaging over the three strongest reflections.

Inductively-coupled plasma-optical emission spectroscopy (ICP-OES). ICP-OES was carried-out by the Microanalysis Services, Department of Chemistry, University of Cambridge using a Thermo Scientific iCAP 7400 spectrometer. Samples were digested in HNO_3 and diluted with ultrapure water to 1-10 ppm analyte. Blank samples of diluted HNO_3 were recorded as background.

External quantum efficiency (EQE). Photocatalysis samples were prepared as stated in the Experimental Section, but using an airtight, flat-sided quartz cuvette (1 cm path length) as the photoreactor. The cuvette was purged with CO_2/CH_4 (2 %) and primed by irradiation for 2 h with a solar light simulator as stated above. The cuvette was then purged again with CO_2/CH_4 (2 %) and irradiated with monochromatic light (λ = 400±5 nm, I = 1.0 mW cm^{-2}, A = 0.25 cm^2) using an LOT Quantum Design MSH-300 monochromator. Aliquots of headspace gas were taken periodically and analysed by gas chromatography. The EQE was calculated according to equation (2).

$$\text{EQE}\ (\%) = \frac{2n \times N_A \times h \times c}{t_{\text{irr}} \times \lambda \times I \times A} \times 100 \qquad (2)$$

Where n is the amount of produced CO or H_2 per time, N_A is Avogadro's constant, h is the Planck constant, c is the speed of light, t_{irr} is the irradiation time, λ is the irradiation wavelength, I is the irradiation intensity and A is the irradiated area.

Gas chromatography analysis. Gas chromatography was carried out on a Shimadzu Tracera GC-2010 Plus gas chromatograph kept at 130 °C using a barrier ionisation discharge (BID) detector and a molsieve column with He as the carrier gas. Methane (2 % CH_4 in CO_2, BOC) was used as internal standard after calibration with different mixtures of known CH_4/H_2/CO compositions.

Infrared spectroscopy. IR spectra were recorded on a Thermo Scientific Nicolet iS50 FT-IR spectrometer. IR spectra of ZnSe-St and ZnSe-BF_4 were recorded in ATR mode by drying one drop of QD stock solution on an FTO-coated glass slide *in vacuo*.

Transmission electron microscopy (TEM). TEM images were collected using an FEI Phillips Technai F20 TEM, operating at an accelerating voltage of 200 kV located at the Electron Microscopy Suite of the Cavendish Laboratory, University of Cambridge.

Zeta potential. Zeta potential measurements of ZnSe-BF_4 (0.5 µM in water, pH adjusted to 5.5 with NaOH/HBF_4) in the presence of varying amounts of MEDA were conducted using a Malvern Zetasizer Nano ZS.

UV–Vis spectroscopy. UV–Vis spectra were recorded on a Varian Cary 50 UV–Vis spectrophotometer using quartz glass cuvettes (1 cm path length).

X-ray photoelectron spectroscopy. XPS spectra were recorded on an ESCALAB 250Xi located at the Optoelectronics group at the Cavendish Laboratory, University of Cambridge, operated by Chris Amey. Samples were prepared by drop-casting stock solutions of QDs on a Cu foil followed by drying in *vacuo*. The background of the spectra was subtracted and the spectra were subsequently fitted using PsdVoigt functions.

Treatment of data. All analytical measurements were performed in triplicate and are given as unweighted mean ± standard deviation (σ) unless otherwise stated. σ of a measured value was calculated using equation (3).

$$\sigma = \sqrt{\frac{\sum(x-\bar{x})^2}{n-1}} \qquad (3)$$

Where n is the number of repeated measurements, x is the value of a single measurement and $\bar{x}$ is the unweighted mean of the measurements. σ was increased to 5 % of $\bar{x}$ in the event that the calculated σ was below this threshold. Lines between data points in Figures 3D, 4B and Figure S4 have been added to guide the eye.

Supporting Tables

Table S1. Attachment of different catalysts on ZnSe-BF_4 based on ion-coupled plasma optical emission spectroscopy (ICP-OES). Samples (0.5 µM QD-BF_4, 10 µM catalyst, in 26 mL 0.1 M aq. AA pH 5.5 under CO_2) were stirred in the dark for 2 h, centrifuged and the precipitate digested in nitric acid.

Catalyst	Zn^{2+} / ppm	Ni^{2+} / ppb	Ni per QD ± σ / mol Ni (mol QD)$^{-1}$
Ni(cyclam)Cl_2	8.946	4.231	0.580±0.03
	11.33	5.769	
NiCycP	9.154	12.69	1.57±0.11
	8.569	10.77	
none	9.077	0.00	0
	9.608	0.00	

Table S2. Optimisation of photocatalytic CO_2 reduction using ZnSe-BF_4/NiCycP. Unless otherwise stated, standard conditions were: 0.5 µM QD, 0.1 M AA, pH 5.5, 2 mL water under CO_2; 100 mW cm^{-2}, AM 1.5G, λ >400 nm, 4 h irradiation, 25 °C.

Co-catalyst	Co-catalyst loading / µM	n(CO) ± σ / µmol	n(H_2) ± σ / µmol	TON_{CO} ± σ / mol CO (mol Ni)$^{-1}$	CO selectivity[a] / %
varying co-catalyst loading					
NiCycP	0	0.153±0.058	12.7±3.0	n/a	1.3±0.9
NiCycP	5	0.829±0.100	12.2±1.4	82.9±10.0	6.4±0.3
NiCycP	10	1.34±0.26	9.71±1.59	66.9±12.7	12.1±1.3
NiCycP	25	1.24±0.54	6.31±2.28	24.8±10.9	16.0±1.6
NiCycP	50	0.973±0.345	4.39±1.34	9.7±3.5	18.0±0.9
NiCycP	150	0.620±0.238	2.26±0.87	2.1±0.8	21.5±0.7
varying the co-catalyst					
NiCycP	10	1.34±0.26	9.71±1.59	66.9±12.7	12.1±1.3
Ni(cyclam)Cl_2	10	0.416±0.088	10.3±1.5	20.8±4.4	3.9±0.5
none	0	0.153±0.058	12.7±3.0	n/a	1.3±0.9

[a] CO selectivity = 100 % × n_{CO} / (n_{CO} + n_{H2}).

Table S3. Control experiments for the photocatalytic CO_2 reduction using ZnSe-BF_4/NiCycP/MEDA. Unless otherwise stated, conditions were: 0.5 µM ZnSe-BF_4, 10 µM NiCycP, 25 µM MEDA, 0.1 M AA, pH 5.5, 2 mL water under CO_2; 100 mW cm^{-2}, AM 1.5G, λ >400 nm, 25 °C.

description	time / h	n(CO) ± σ / µmol	n(H_2) ± σ / µmol
standard experiment	20	5.66±0.47	11.1±0.7
no AA	20	0.0257	not detected
no light	20	not detected	not detected
no ZnSe-BF_4	17	not detected	not detected
no NiCycP	20	0.652±0.070[a]	15.7±2.4[a]

[a] Data from two independent experiments.

Table S4. Optimisation of photocatalytic CO_2 reduction using ZnSe-BF_4/NiCycP in the presence of MEDA. Conditions: 0.5 µM ZnSe-BF_4, 10 µM NiCycP, 0.1 M AA, pH 5.5, 2 mL water under CO_2; 100 mW cm^{-2}, AM 1.5G, λ >400 nm, 4 h irradiation, 25 °C.

MEDA [µM]	n(CO) ± σ / µmol	n(H_2) ± σ / µmol	TON_{CO} ± σ / mol CO (mol Ni)$^{-1}$	CO selectivity[a] / %
0	1.34±0.26	9.71±1.59	66.9±12.7	12.1±1.3
12.5	2.34±0.78	5.85±1.46	117±39	28.2±2.8
25	1.92±0.24	4.60±0.78	96.1±12.2	29.6±1.5
37.5	1.81±0.50	4.28±1.08	90.4±25.1	29.6±2.3
50	0.971±0.431	3.21±1.15	48.6±21.6	22.7±2.2
75	0.855±0.334	3.05±0.88	42.8±16.7	21.6±2.0
100	0.622±0.222	2.39±0.89	31.1±11.1	20.7±1.2

[a] CO selectivity = 100 % × n_{CO} / (n_{CO} + n_{H2}).

Table S5. Zeta potential measurements of ZnSe-BF_4 in the presence of MEDA (0.5 µM ZnSe-BF_4 in 2 mL water, pH 5.5, rt).

c (MEDA) / µM	Zeta potential / mV
0	3.28±0.06
25	18.1±0.9
100	23.9±0.5

Table S6. External quantum efficiency (EQE) determination for the photocatalytic CO_2 reduction using Zn-Se-BF_4/NiCycP/MEDA (1.0 µM ZnSe-BF_4, 20 µM NiCycP, 50 µM MEDA in 2 mL 0.1 M aq. AA, pH 5.5 under CO_2; I = 1.00 mW cm^{-2}, A = 0.25 cm^2, λ = 400±5 nm, rt).

time / h	n(CO) / nmol[a]	EQE_{CO} / %[b]	n(H_2) / nmol[a]	EQE_{H2} / %[b]
2	111±6	3.75±0.19	135±7	4.55±0.23
4	210±11	3.34±0.17	257±13	4.14±0.21
6	301±15	3.08±0.15	376±19	4.00±0.20
20	792±65	2.35±0.28	989±50	2.93±0.18
average over the first 6 h		3.39±0.30		4.23±0.24

[a] Cumulative product measured in headspace.
[b] Quantum efficiency measured per time interval.

Table S7. Summary of fitting parameters obtained from the kinetic traces of the main features in the TAS spectra. Spectra were recorded with aqueous ZnSe-BF_4 (0.5 µM) at pH 6.5, with or without AA (0.1 M), with or without MEDA (25 µM), with or without NiCycP (10 µM) unless otherwise stated.

Sample	magnitude ± σ	lifetime ± σ / ps	rate ± σ / s^{-1}
(425 nm) ZnSe-BF_4	A_1: –7.1(±1)x10^{-3}	τ_1: 0.9(±0.2)	k_1: 1.1(±0.2)
	A_2: –6.9(±1.4)x10^{-4}	τ_2: 35(±15)	k_2: 2.8(±1.2)x10^{-2}
(470 nm) ZnSe-BF_4	A_1: 6.2(±0.4)x10^{-4}	τ_1: 25(±4)	k_1: 4.0(±0.7)x10^{-2}
	A_2: 2.9(±0.4)x10^{-4}	τ_2: 409(±106)	k_2: 2.4 (±0.6)x10^{-3}
(590 nm) ZnSe-BF_4	A_1: 2.9(±1.7)x10^{-4}	τ_1: 21(±6)	k_1: 4.7(±1.0)x10^{-2}
	A_2: 1.9(±0.2)x10^{-4}	τ_2: 475(±72)	k_2: 2.1 (±0.3)x10^{-3}
(580 nm) ZnSe-BF_4	A_1: 4.1(±0.3)x10^{-4}	τ_1: 5.7(±0.7)	k_1: 1.7(±0.2)x10^{-1}
	A_2: 2.48(±0.08)x10^{-4}	τ_2: 290(±106)	k_2: 3.4 (±0.2)x10^{-3}
(520 nm) ZnSe-BF_4/AA	A_1: –1.06(±0.06)x10^{-3}	τ_1: 3.7(±0.4)	k_1: 2.7(±0.3)x10^{-1}
	A_2: –5.8(±0.3)x10^{-4}	τ_2: 113(±16)	k_2: 8.8 (±1.2)x10^{-3}
	A_3: –5.3(±0.6)x10^{-4}	τ_3: 2307(±790)	k_3: 4.3 (±1.4)x10^{-4}
(520 nm) ZnSe-BF_4/AA/NiCycP	A_1: –1.3(±0.3)x10^{-4}	τ_1: 1.3(±0.3)	k_1: 7.4(±1.9)x10^{-1}
	A_2: –7.0(±0.4)x10^{-4}	τ_2: 47(±6)	k_2: 2.1(±0.3)x10^{-2}
	A_3: –4.6(±0.2)x10^{-4}	τ_3: 910(±161)	k_3: 1.1 (±0.2)x10^{-4}

Supporting Figures

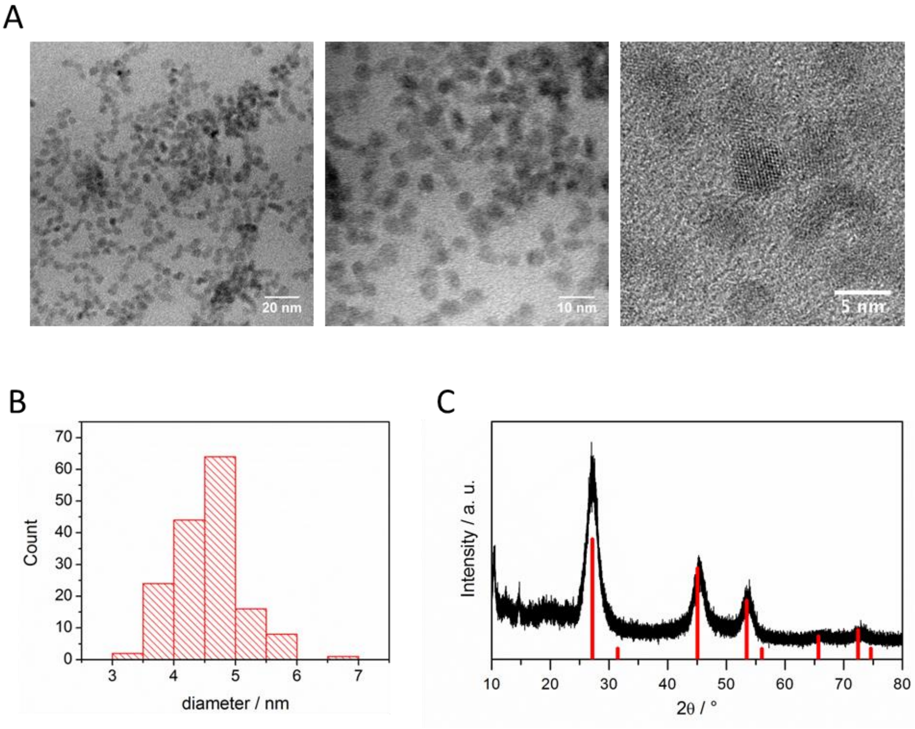

Figure S1. Characterisation of stearate-capped ZnSe quantum dots (ZnSe-St): A) Transmission electron micrographs; B) particle size distribution determined by TEM; C) powder X-ray diffractogram overlaid with cubic zinc blende ZnSe reference (PDF 01-071-5978).

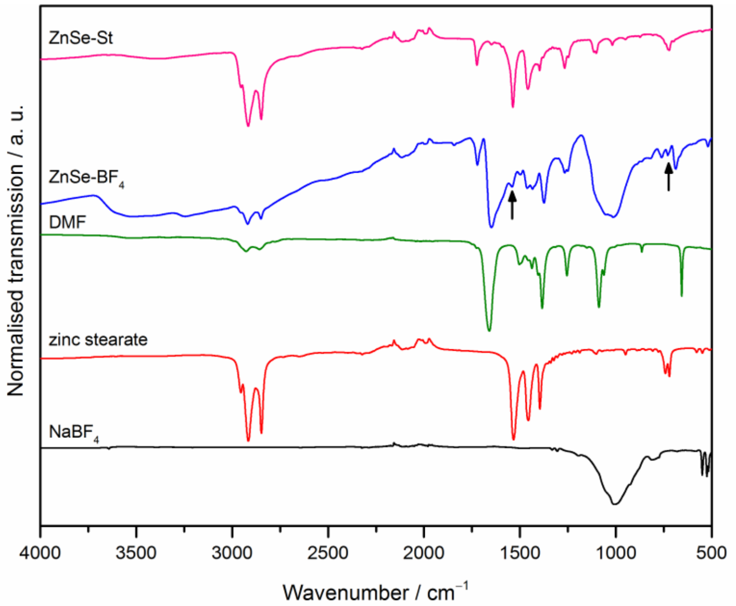

Figure S2. ATR-IR spectra of ZnSe quantum dots before (ZnSe-St) and after stripping (ZnSe-BF_4) and comparison with the spectra of DMF, zinc stearate and $NaBF_4$. Signals assigned to residual stearate on ZnSe-BF_4 are highlighted with black arrows.

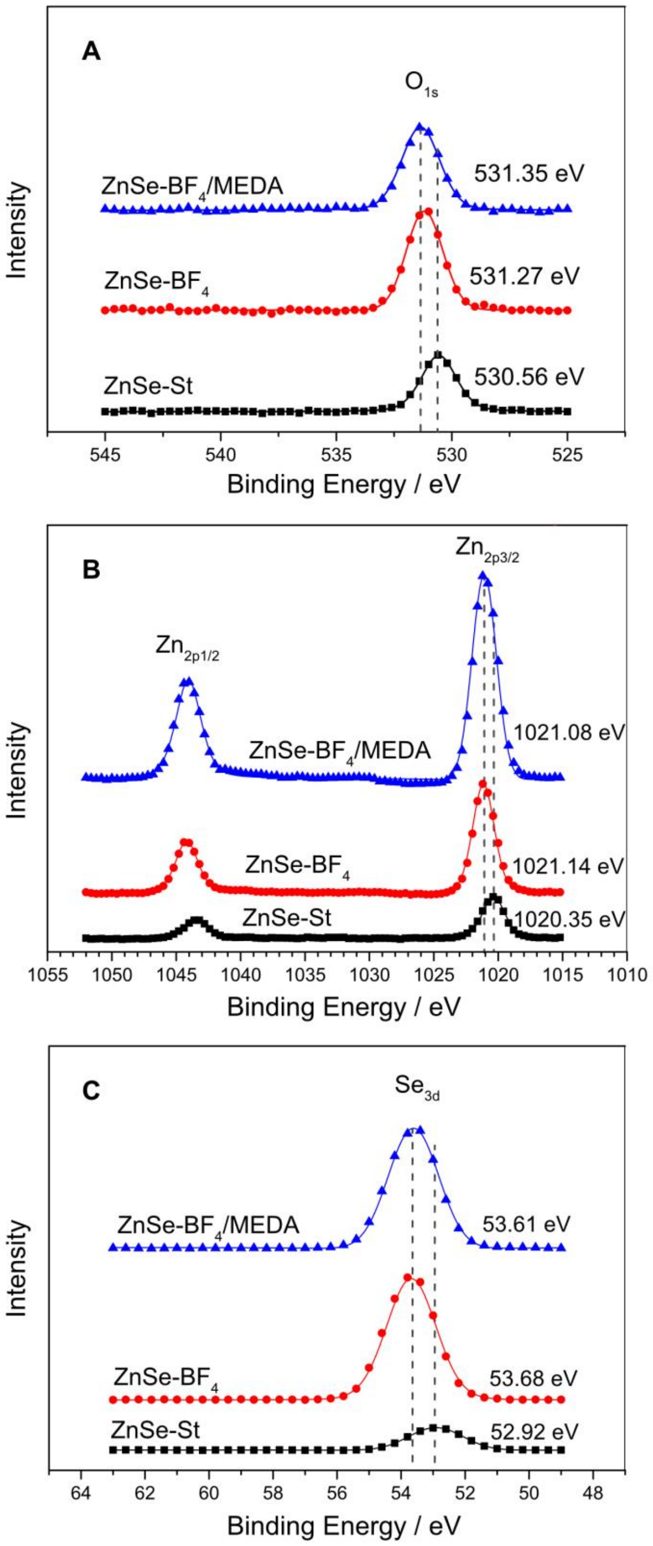

Figure S3. (A) O_{1s}, (B) Zn_{2p} and (C) Se_{3d} regions of XPS spectra of ZnSe quantum dots before (ZnSe-St) and after ligand stripping (ZnSe-BF_4), and in the presence of MEDA (ZnSe-BF_4/MEDA).

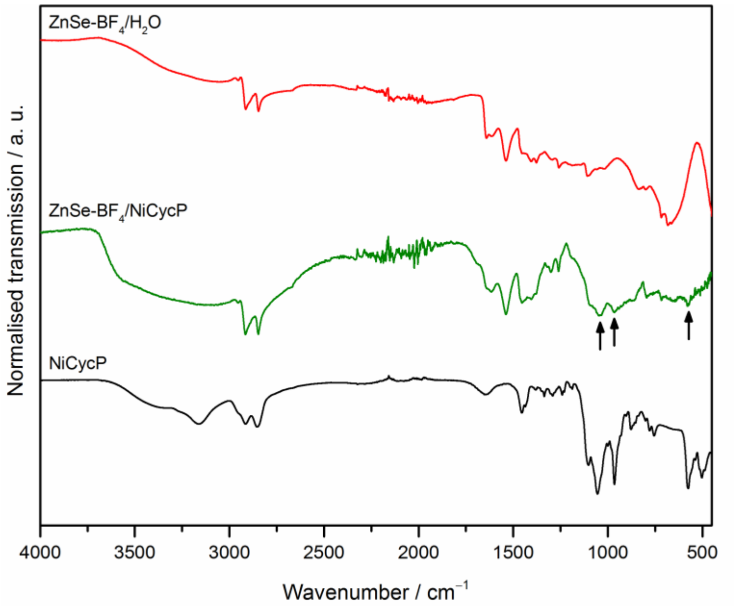

Figure S4. ATR-IR spectra of $ZnSe-BF_4$ modified with NiCycP. $ZnSe-BF_4$ QDs were incubated in aqueous NiCycP, washed with water to remove excess NiCycP and dried. Vertical arrows indicate bands assigned to adsorbed NiCycP by comparison with the spectra of blank $ZnSe-BF_4$ and neat NiCycP. Note the absence of B-F stretches (expected around 1000 cm^{-1}, cf. Figure S2), upon incubation of $ZnSe-BF_4$ in water.

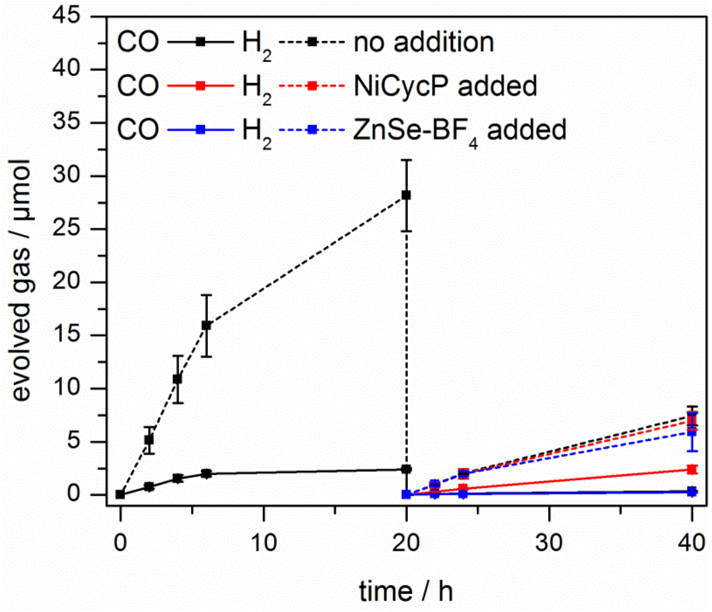

Figure S5. Long-term photocatalytic activity of ZnSe-BF_4/NiCycP. Samples were re-purged with CO_2 after 20 h and 0.5 µM ZnSe-BF_4, 10 µM NiCycP or nothing was added before irradiation was continued (0.5 µM QD, 10 µM NiCycP in 0.1 M aq. AA, pH 5.5 under CO_2, 100 mW cm^{-2}, AM 1.5G, λ >400 nm, 25 °C).

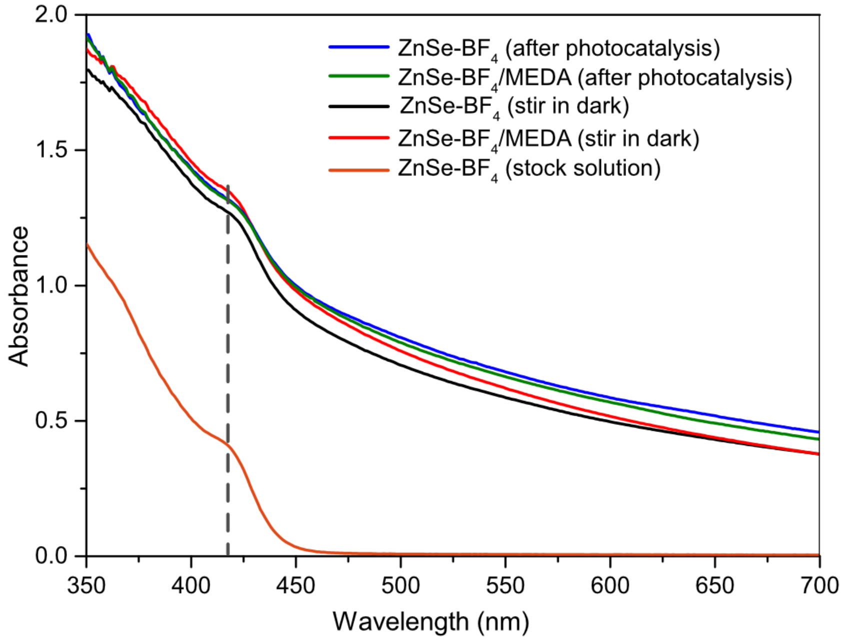

Figure S6. UV-vis characterisation of the photosystem after irradiation for 20 h. Samples in the presence and absence of MEDA are compared to samples which have been stirred in the dark for 20 h under otherwise similar conditions. Comparison of a ZnSe-BF_4 stock solution in DMF is given. Conditions: 1 µM QD, 20 µM NiCycP in 0.1 M aq. AA, 50 µM MEDA, pH 5.5 under CO_2; 20 h irradiation, 100 mW cm^{-2}, AM 1.5G, λ >400 nm, 25 °C.

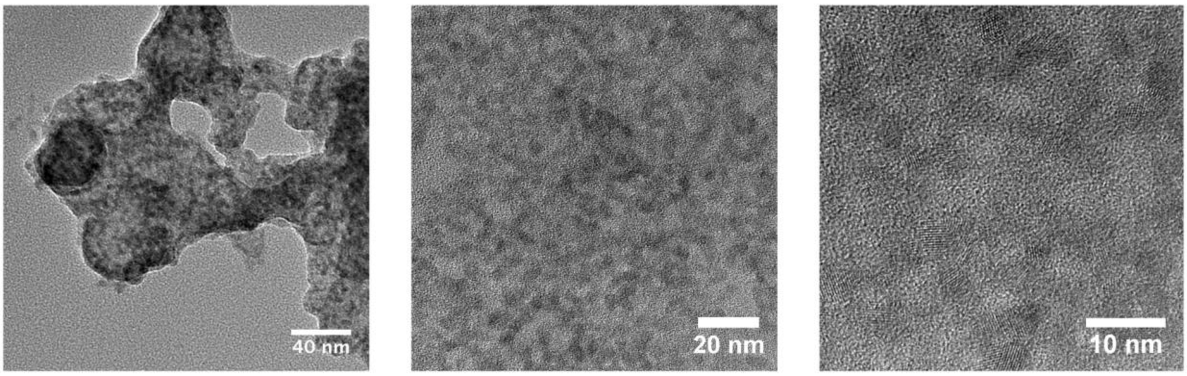

Figure S7. Transmission electron micrographs of ZnSe-BF_4-QDs after irradiation for 4 h showing aggregated structures and well-dispersed particles. The nanocrystalline fine structure remains clearly visible. Particles were precipitated via centrifugation after photocatalysis. Conditions: 0.5 µM QD, 10 µM NiCycP in 0.1 M aq. AA, 25 µM MEDA, pH 5.5 under CO_2; 4 h irradiation, 100 mW cm^{-2}, AM 1.5G, λ >400 nm, 25 °C

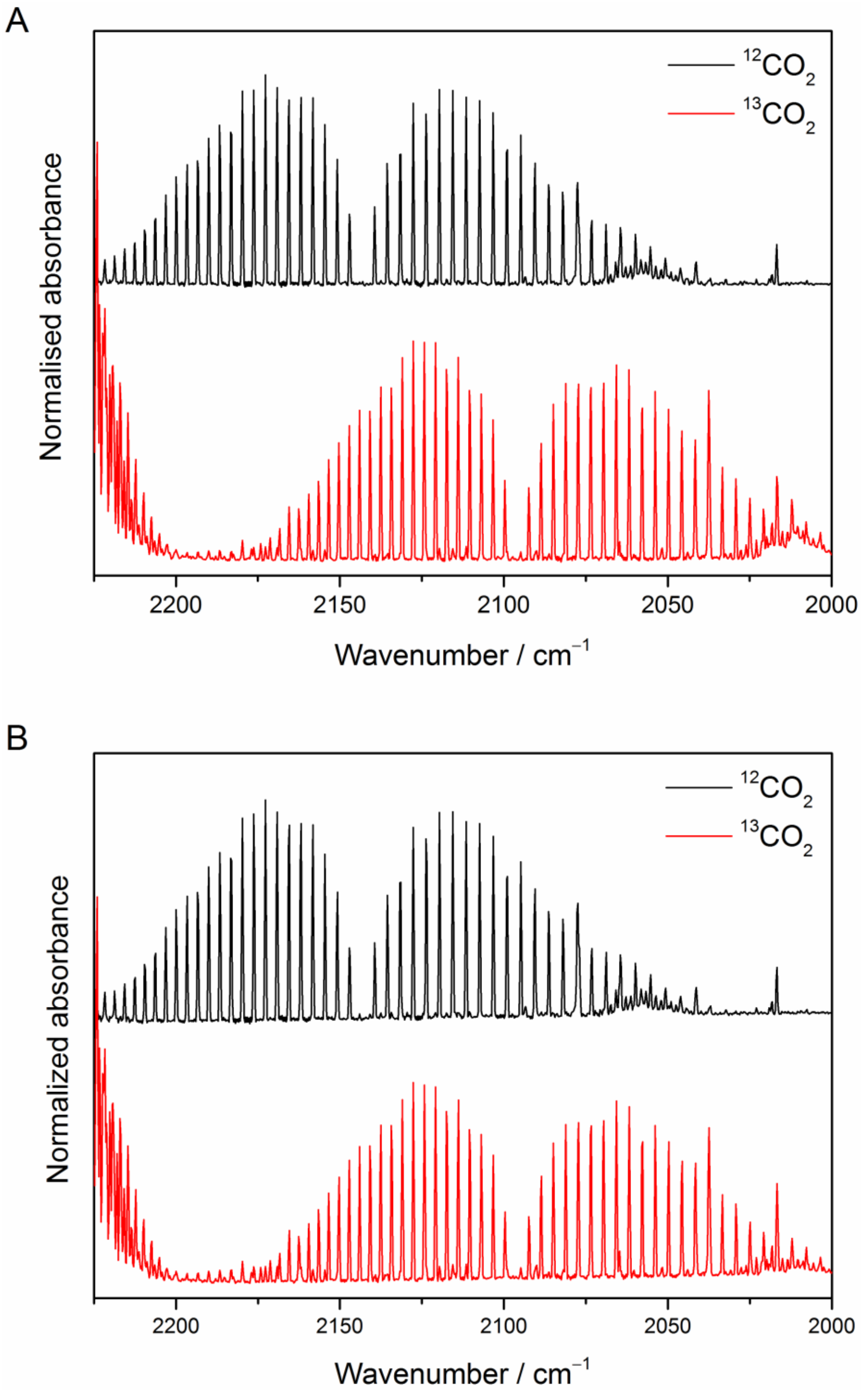

Figure S8. Gas-phase transmission IR spectra of the photocatalytic CO_2 reduction products depending on the employed CO_2 isotopologue. A) In the absence of MEDA; B) in the presence of 25 µM MEDA (0.5 µM QD, 10 µM NiCycP in 0.1 M aq. AA, pH 5.5 under $^{12}CO_2$ or $^{13}CO_2$; 15 h irradiation, 100 mW cm^{-2}, AM 1.5G, λ >400 nm, 25 °C).

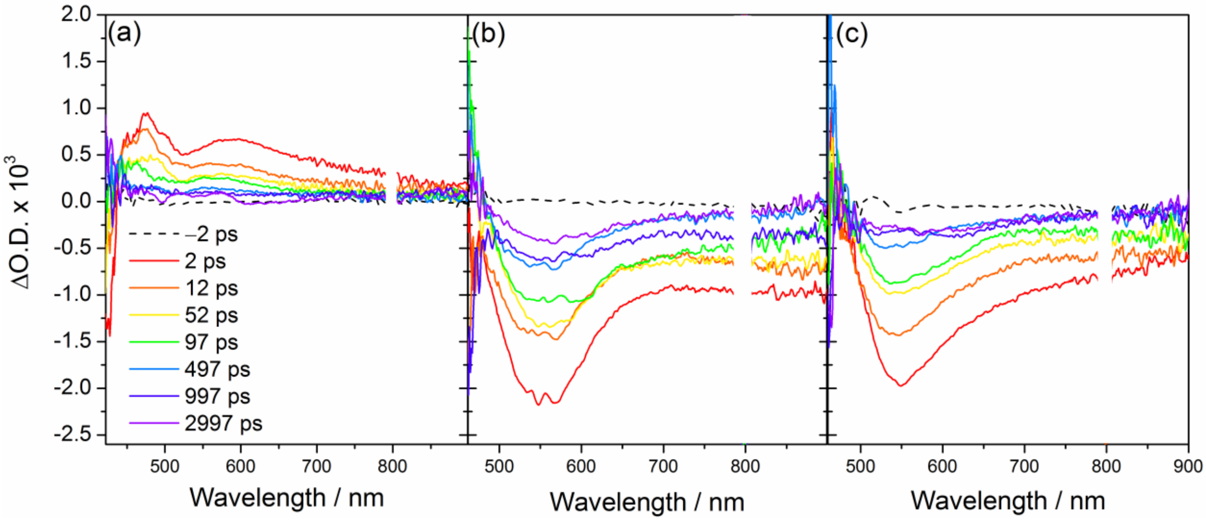

Figure S9. TA spectroscopy of the ZnSe-BF_4/NiCycP/MEDA photocatalyst under different conditions: Band gap excitation of ZnSe-BF_4/MEDA (A) in the absence of AA produces a ground state bleach and a positive feature (hole), (B) in the presence of AA produces a long-lived red-shifted bleach (trapped electrons), and (C) in the presence of NiCycP and AA accelerates recovery of the trap state bleach (400 nm excitation, 450-900 nm probe).

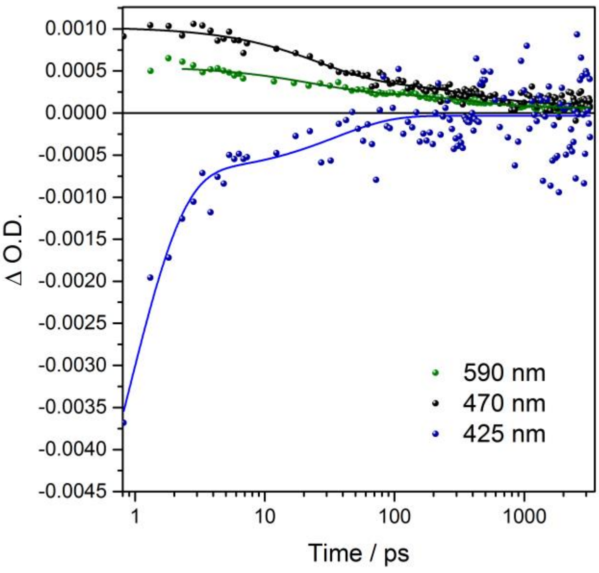

Figure S10. TAS kinetics at indicated wavelength of ZnSe-BF_4/MEDA (0.5 µM ZnSe-BF_4, 25 µM MEDA in H_2O) in the absence of AA following 400 nm excitation. The solid lines are from the biexponential fit of the data, see table S7 for fitting parameters.

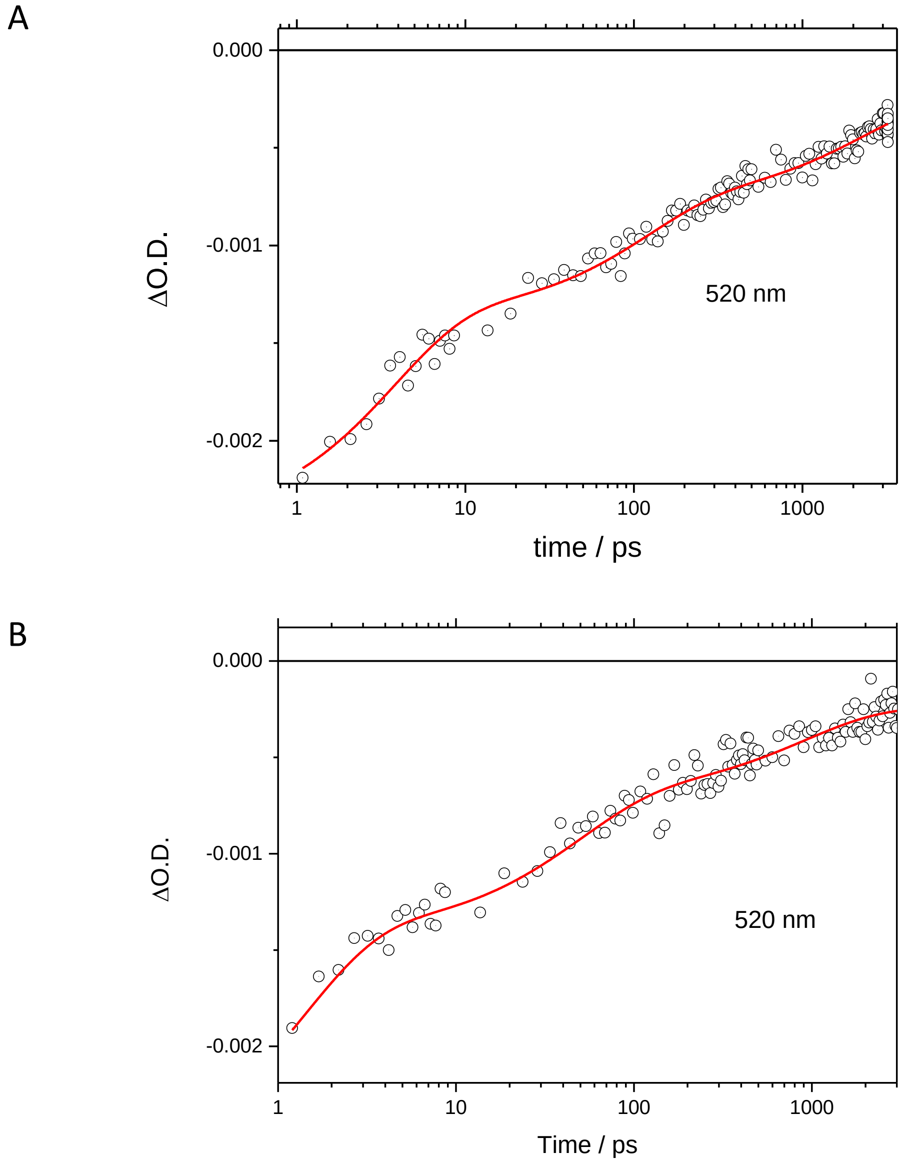

Figure S11. TAS kinetics of ZnSe-BF_4/MEDA (0.5 µM ZnSe-BF_4, 25 µM MEDA) in aqueous solution in the presence of AA (0.1 M, pH 6.5) thoroughly purged with argon. A) In the absence of NiCycP; B) in the presence of NiCycP (10 µM). The solid red lines are from fitting to a triexponential function, see text below.

The recovery of the bleach signal assigned to the trapped photoelectrons can be reasonably fitted to a minimum of a triexponential function (eq. 4).

$$(y = y_0 + A_1 e^{\frac{-x}{\tau_1}} + A_2 e^{\frac{-x}{\tau_2}} + A_3 e^{\frac{-x}{\tau_3}}) \qquad (4)$$

In the absence of NiCycP, τ_1 = 3.5±0.4 ps, A_1 = −0.011, τ_2 = 113±16 ps, A_2 = −0.006, τ_3 = 2310±790 ps, A_4 = −0.005, in the presence of NiCycP, τ_1 = 1.4±0.4 ps, A_1 = −0.011, τ_2 = 47±6 ps, A_2 = −0.007, τ_3 = 910±160 ps, A_3 = −0.005. In both cases, a residual bleach (y_0 = −2.4×10^{-3}) persists in the fits and it can be seen in the TAS data that a weak signal is still present at 520 nm at the longest time after excitation that can be studied by this apparatus (3 ns). Attempts to identify the signature of trapped electrons using slower transient absorption spectroscopy apparatus (maximum time resolution *ca.* 1 µs) were unsuccessful.

Supporting references

(1) Borchert, H.; Shevchenko, E. V.; Robert, A.; Mekis, I.; Kornowski, A.; Grübel, G.; Weller, H. *Langmuir* **2005**, *21*, 1931-1936.

(2) (a) Othonos, A.; Lioudakis, E.; Philipose, U.; Ruda, H. E. *Appl. Phys. Lett.* **2007**, *91*, 241113; (b) Matylitsky, V. V.; Shavel, A.; Gaponik, N.; Eychmüller, A.; Wachtveitl, J. *J. Phys. Chem. C* **2008**, *112*, 2703-2710.
